# Assessment of Serum Progesterone Level on the Day of hCG Injection in Infertile Polycystic Ovarian Syndrome Patients Referred to Women’s Hospital, Tehran, 2009

**Published:** 2012-03-20

**Authors:** Zahra Rezaee, Azizeh Ghaseminejad, Mitra Forootan, Taraneh Hosseinipoor, Forough Forghani

**Affiliations:** 1 Obstetrics and Gynecology Department, Tehran University of Medical Sciences, Tehran, Iran; 2 Zabol University of Medical Sciences, Zabol, Iran

**Keywords:** Progesterone, Infertility, PCOS, HCG

## Abstract

**Background:**

Polycystic ovarian syndrome (PCOS) is one of the most common causes of endocrine disorders and main reasons for infertility due to unovulation and recurrent abortions. There is no consensus on effect of serum progesterone level on the day of human chorionic gonadotropin (hCG) injection. This study aims to evaluate the effect of plasma levels of progesterone on the day of hCG injection on the rate of pregnancy in *in vitro* fertilization (IVF) cycles of PCOS cases.

**Materials and Methods:**

A stratified cohort study was conducted over a period of one year (2009) on 38 infertile women with PCOS who were suitable candidates for the IVF program. Patients were evaluated for other causes of infertility with hysterosalpingography (HSG), laparoscopy and normal sperm analysis. Patients were placed on the long protocol, followed by oocyte pick up, and finally IVF-embryo transfer (ET). Study patients were grouped according to progesterone levels of greater or less than 1.2 ng/ml on the day of hCG injection. Pregnancy rates were defined in each group. Levels on day of hCG day clinical pregnancy outcome were assessed. Experimental data were then compared against Fisher’s exact test in SPSS version 18.

**Results:**

The overall pregnancy rate in this study was 26.3%. In the group with progesterone levels more than 1.2 ng/ml on the day of hCG injection, the clinical pregnancy rate was 4 (21.1%) and chemical pregnancy rate was 3(15.8%). In the group with progesterone levels less than 1.2 ng/ml, the clinical pregnancy rate was 1(5.3%) and chemical pregnancy rate was 2(10. 5%).

**Conclusion:**

This study showed that PCOS patients with progesterone levels more than 1.2 ng/ml on the day of hCG injection resulted in higher chemical and clinical pregnancy rates. However, no significant statistical differences were found between the two groups. For further verification, we recommend additional studies with larger numbers of subjects.

## Introduction

Polycystic ovarian syndrome (PCOS) is one of the most common causes of endocrine disorders and main reasons forinfertility due to unovulation and recurrent abortions (1). Clomiphene citrate is the initial treatment for most unovulatory infertile women. However, the pregnancy rate is disappointingly lower than expected (50% or less). Individuals not responding to this treatment are often called clomiphene citrate resistant (CCR). An alternative medication for CCR is the administration of exogenous gonadotropin by injection. If this is not successful, the treatment for such cases is *in vitro* fertilization (IVF) (2). Most often, GnRH analogs are used in conjunction with gonadotropin to prevent the possibility of spontaneous luteinizing hormone (LH) surge and ovulation prior to egg retrieval. It is reported that, despite an effective suppression of endogenous gonadotropin by GnRH analog, there is a small increase in plasma progesterone in up to 20% of stimulated cycles (3). Thus, the issue of a potential adverse effect of progesterone increase on the cycle outcome must be addressed. However, it is still a matter of debate. While some authors report a negative effect on the pregnancy rate when the plasma level of progesterone is more than 0.9 ng/ml on the day of human chorionic gonadotropin (hCG) injection in IVF cycles (4), others such as Hoffman et al. (5,6) did not find any significant relationship. These researchers have also stated that the high level of plasma progesterone on the day of hCG administration on ovum donors has no adverse effect on pregnancy rate. In 1993, Lergo et al. (7) showed that premature LH surge with elevated progesterone levels increased pregnancy rates of ovum donors during IVF cycles. The aim of this study was to evaluate the effect of plasma progesterone levels on the day of hCG administration on clinical pregnancy rates in IVF cycles of PCOS patients.

## Materials and Methods

This stratified cohort study was conducted over a period of one year (2009) on 38 infertile women with PCOS who were suitable candidates for the IVF program. All cases were between ages 20 to 38 years. Patients were evaluated for other causes of infertility by hysterosalpingography (HSG) and laparoscopy. Their partners' sperm analyses, according to WHO guidelines (2002) were all normal. The criteria for selection of PCOS patients were based on: i. history of amenorrhea or oligomenorrhea; ii. hyperandrogenism (i.e., hirsutism >7 according to Ferriman Gallweg score ; iii. elevated LH or LH/follicle stimulating hormone (FSH) >2; and iv. increased ovarian volume >9 ml or antral follicle count ≥10 as seen with ultrasound.

All patients underwent the long pituitary down regulation protocol. They were given low doses of oral contraceptives (OCP) on the third day of menstruation. On days 20 to 21 of their menstrual cycles (late luteal phase), a GnRH agonist (Suprefact, Hoechst, Aquila, Italy) at 500 ìg/day, sub-cutaneous injection was begun.

After two weeks of Suprefact injections, on the third day of the next menstrual cycle, serum estradiol levels were checked and a vaginal sonographic scan was performed to confirm pituitary suppression. Induction ovulation was started in cases where: i. serum estradiol levels were <50 pg/ml, ii. there was no cyst or follicle >10 mm, and iii. the endometrial thickness was <4 mm. Otherwise, GnRH agonist would be given for an extra week, for up to 21 days. On the third day of menstruation, 150-225 units of gonadotropin[rFSH (gonal-F; Serono, Aubonne, Switzerland)] was administered according to age, weight, and FSH levels. Suprefact was decreased to half of its initial dose on that day. After administrating gonal-F for 5 days, ovarian response was monitored by vaginal ultrasound; then gonal-F was regulated. When there were at least two 18 mm follicles, hCG (Profasi, Serono, Rome, Italy) at a dose of 10000 IU was injected. The progesterone level was checked on the day of hCG injection. Oocyte pickup was performed 36-38 hours later. After a period of 48 hours, embryo transfer with a Cook catheter (Cook catheter OB/GYN spencer JN) was performed. There were between 2-4 embryos that were transferred, dependent upon the embryo age and quality. Luteal phase support was carried out by progesterone injection, 100 mg per day from the oocyte retrieval day.

Subjects were selected through interviews, examinations, and lab tests. Upon receiving information, all subjects consented to participate in the study. According to serum progesterone levels on the day of hCG injection, patients were divided into 2 groups of: i. <1.2 ng/ml or ii. >1.2 ng/ml. Clinical pregnancy was defined as detection of the fetal heart by ultrasound at 6-8 weeks gestation Chemical pregnancy was defined as ß hCG levels >200 IU. The rate of pregnancy was compared between the two groups. The experimental data were then compared against Fisher’s exact test (SPSS, version 18). The level of significance was considered based on the criteria of p<0.05. This survey was conducted as an obstetrics and gynecology educational thesis at Tehran University of Medical Sciences and patients incurred no charges.

## Results

A total of 38 PCOS patients participated in this study. Of these, 30 (78.9%) had primary infertility. The mean infertility duration was 8.7 ± 3.5 years and mean age of the patients was 34.7 ± 4.5 years. Mean basal serum FSH level was 7.4 ± 2.6 mIU/ mL and E2 level was 59.2 ± 30.8 pg/mL.

The mean total dosage of gonadotropin used was 1950.8 ± 500.0 IU mean duration of induction was 13.1 ± 2.2 days. The mean number of oocytes was 10.4 ± 2.2 and mean total number of embryos was 8.2 ± 2.8.

Overall, the pregnancy rate in this study was 10 (26.3%). As stated earlier, the 38 PCOS patients considered for this study were divided into 2 groups based on progesterone levels on the day of hCG injection in IVF cycles. The findings of this experiment are summarized in table 1.

**Table 1:** Comparison of pregnancy outcome in PCOS patients according to progesterone level


Group / pregnancy result	Pregnancy rate	Clinical pregnancy	Chemical pregnancy

**Progesterone <1.2**	3 (15.8%)	1 (5.3%)	2 (10.5%)
**Progesterone ≥1.2**	7 (36.84%)	4 (21.1%)	3 (15.8%)
**P value**	0.27	0.34	0.34


## Discussion

This study showed that clinical and chemical pregnancy outcomes in PCOS patients with progesterone levels above 1.2 ng/ml were higher than those whose levels were less than 1.2 ng/ml. However, no significant statistical differences were found.

In two separate studies, Hoffman et al. (5, 6) showed no statistical differences between patients with IVF cycles according to serum progesterone levels on the day of hCG injection, even in donor cases. Huang et al. (8) reported no differences in clinical pregnancy rate between 3 groups of patients with progesterone levels less than 0.3 ng/ml, between 0.3 and 1 ng/ml, and more than 1 ng/ml on the day of hCG injection in IVF cycles. Silverberg and coworkers (4) have shown that increasing serum progesterone levels on the day of hCG injection had no adverse effect on oocyte quality. Abuzeid and colleagues (9) showed that the level of progesterone on the day of hCG injection had no negative effect on pregnancy rate, whereas Bustillo et al. (10) cited that the level of progesterone on the day of hCG injection was not predictive of pregnancy rate. They have also shown that premature luteinization with high progesterone levels is a common event in IVF cycles. It is a natural sign of follicular development, therefore it increases pregnancy rates. Similarly, Doldi et al. (2) have reported that premature increases of serum progesterone in IVF PCOS cases could be a predictive factor of IVF success rate. In contrast, however, other studies have found that Explained relation between premature luteinization and low pregnancy rate in IVF cycles.Schoolcraft and colleagues (11) have shown that serum progesterone levels above 0.5 ng/ml on the day of hCG injection had a significant negative correlation with pregnancy rate in IVF cycles. Harada et al. (12) cited that a small increase in serum progesterone on the day of hCG injection inversely affected IVF cycles. Burns et al. (13) proved that there was a negative correlation between the low pregnancy rate in IVF cycles and premature increase in serum progesterone. Dirnfeld and collaborators (14) deduced that the pregnancy rate in IVF cycles declined if there was a small increase in serum progesterone level on the day of hCG injection, which was also confirmed by Mio and coworkers (15).

## Conclusion

It appears that the results of various studies are in fact quite different. These observations or contradictory reports are related to many factors that affect IVF outcome. Induction ovulation is one factor which influences the quality of oocytes and implantation. Therefore, evaluation of serum progesterone levels before oocyte aspiration is complicated. Age, predisposing factors for IVF, and numbers of embryos also change IVF cycle results. The level of serum progesterone selected in various studies is not unanimous and therefore may affect the results.

In conclusion, we recommend studies with more patients, taking into account the bias variable.
